# Proteome profiling of triple negative breast cancer cells overexpressing NOD1 and NOD2 receptors unveils molecular signatures of malignant cell proliferation

**DOI:** 10.1186/s12864-019-5523-6

**Published:** 2019-02-21

**Authors:** Fernando J. Velloso, Alexandre R. Campos, Mari C. Sogayar, Ricardo G. Correa

**Affiliations:** 1 Cell and Molecular Therapy Center (NUCEL), Internal Medicine Department, School of Medicine, University of São Paulo (USP), São Paulo, SP 05360-130 Brazil; 2 SBP Medical Discovery Institute, 10901 North Torrey Pines Rd, La Jolla, CA 92037 USA

**Keywords:** NOD1, NOD2, Proteome, NLR, Hs578T, NF-κB, MAPK triple negative breast cancer

## Abstract

**Background:**

Triple negative breast cancer (TNBC) is a malignancy with very poor prognosis, due to its aggressive clinical characteristics and lack of response to receptor-targeted drug therapy. In TNBC, immune-related pathways are typically upregulated and may be associated with a better prognosis of the disease, encouraging the pursuit for immunotherapeutic options. A number of immune-related molecules have already been associated to the onset and progression of breast cancer, including NOD1 and NOD2, innate immune receptors of bacterial-derived components which activate pro-inflammatory and survival pathways. In the context of TNBC, overexpression of either *NOD1*or *NOD2* is shown to reduce cell proliferation and increase clonogenic potential in vitro. To further investigate the pathways linking NOD1 and NOD2 signaling to tumorigenesis in TNBC, we undertook a global proteome profiling of TNBC-derived cells ectopically expressing each one of these NOD receptors.

**Results:**

We have identified a total of 95 and 58 differentially regulated proteins in *NOD1*- and *NOD2*-overexpressing cells, respectively. We used bioinformatics analyses to identify enriched molecular signatures aiming to integrate the differentially regulated proteins into functional networks. These analyses suggest that overexpression of both *NOD1* and *NOD2* may disrupt immune-related pathways, particularly NF-κB and MAPK signaling cascades. Moreover, overexpression of either of these receptors may affect several stress response and protein degradation systems, such as autophagy and the ubiquitin-proteasome complex. Interestingly, the levels of several proteins associated to cellular adhesion and migration were also affected in these NOD-overexpressing cells.

**Conclusions:**

Our proteomic analyses shed new light on the molecular pathways that may be modulating tumorigenesis via NOD1 and NOD2 signaling in TNBC. Up- and downregulation of several proteins associated to inflammation and stress response pathways may promote activation of protein degradation systems, as well as modulate cell-cycle and cellular adhesion proteins. Altogether, these signals seem to be modulating cellular proliferation and migration via NF-κB, PI3K/Akt/mTOR and MAPK signaling pathways. Further investigation of altered proteins in these pathways may provide more insights on relevant targets, possibly enabling the immunomodulation of tumorigenesis in the aggressive TNBC phenotype.

**Electronic supplementary material:**

The online version of this article (10.1186/s12864-019-5523-6) contains supplementary material, which is available to authorized users.

## Background

Breast cancer is the most common type of non-epidermal cancer in women, accounting for 25% of all female cancers diagnosed, being the leading cause of cancer mortality, representing 15% of cancer-related deaths in women worldwide [1, 2]. Social changes in lifestyle, reproductive and dietary habits are the main factors driving an increase in breast cancer incidence worldwide, particularly in developing countries [1]. Although early diagnosis through population-based screening and more effective treatment regimens have led to a decline in mortality rates (especially in more developed countries), some types of breast cancer still have very poor prognosis [3, 4], mostly due to the co-emergence of metastatic tumors [5].

Traditionally, breast cancer classification is based on immunohistochemical detection of hallmark proteins associated with cell functions, including receptors for Estrogen (ER), Progesterone (PR) and amplification of HER2 (Human Epidermal Growth Factor Receptor 2) [6]. Approximately 15% of all breast tumors derive from cells of basal origin (basal-like) and lack expression of ER, PR and amplification of HER2, being therefore classified as Triple Negative Breast Cancers (TNBC) [6–8]. TNBC has one of the poorest prognosis among all breast cancers, due to its aggressive clinical characteristics and, more specifically, lack of response to hormonal (ER and PR) or HER2 receptor-targeted drug therapy [7, 9].

Several critical signaling pathways are deregulated during breast cancer progression [6], including immune-related cascades, which may promote tumorigenesis through chronic inflammation [10]. Immune-related genes and pathways are more highly expressed in TNBC than in other breast cancer subtypes [11], suggesting a stronger immunogenicity compared to non-TNBC. Moreover, overexpression of immune-related genes may be correlated with a better prognosis in TNBC [11], encouraging the pursuit of immunotherapeutic options for TNBC.

A number of immune-related molecules have already been associated to the onset and progression of breast cancer, including interleukins, caspases and immune receptors, such as the NLRs (NACHT and Leucine Rich Repeat domain containing proteins) [12–14]. The NLRs recognize both pathogen-associated molecular patterns (PAMPs) and danger associated molecular patterns (DAMPs), acting as innate immunity “sensors” towards pathogen-derived components and cellular damage/stress [15]. Two major NLRs, namely, NOD1 and NOD2 (Nucleotide-Binding Oligomerization Domain-Containing Protein 1 and 2) directly bind to ligands through their variable tandem C-terminal Leucine-Rich Repeat domains (LRRs), which allow these receptors to detect the bacterial peptidoglycans (PGN) iE-DAP (gamma-D-glutamyl-meso-diaminopimelic acid) and MDP (muramyl dipeptide), respectively [16, 17]. In the cytosol, NOD1 and NOD2 are bound to the membranes of early endosomes and interact with the actin cytoskeleton, which helps to keep both receptors in an inactive state and enables receptor re-localization to sites of bacterial entry [18, 19]. After endocytosis [20, 21], PGNs are transported through the endosomal membrane by oligopeptide transporters SLC15A3, SLC15A4 or SLC46A2 [22–25], being promptly recognized by NOD1 and NOD2 receptors. Ligand-bound NOD1 and NOD2 self-oligomerize, using the endosomal membrane as a scaffold for the assembly of signaling complexes [23, 26], and recruits RIPK2 (receptor-interacting serine/threonine-protein kinase 2). RIPK2 is then poly-ubiquitinated by E3 ligases, including TNF receptor-associated factors (TRAFs) [27, 28], bringing together and activating members of the Inhibitory κB Kinase (IKK) complex and TGF-β-activated kinase 1 (TAK1) [29]. TAK1 is a bifurcation point in NOD signaling, interacting with the IKK complex [30], which leads to NF-κB activation through poly-ubiquitination and proteasomal degradation of its inhibitors (IκBs) [31], and also binding to p38 and JNK [30, 32], thus activating stress kinase cascades through MAPKs [33].

NOD1 and NOD2 receptors also respond to bacterial infections through an alternative pathway, independent of RIPK2 and NF-κB signaling. During invasion by intracellular bacteria, NOD1 and NOD2 directly bind and recruit the critical autophagic protein ATG16L1 to the plasma membrane at the bacterial entry site, promoting highly specific segregation and degradation of bacteria by the autophagy machinery [34–37].

In addition to their role as sensors of bacterial derivates, NOD1 and NOD2 receptors also monitor the intracellular environment, responding to perturbations in the actin cytoskeleton and to endoplasmic reticulum (ER) stress [38, 39]. ER stress elicits the unfolded protein response (UPR) system, which increases expression of chaperones and modifying enzymes needed to properly fold proteins and, ultimately, activates autophagy [40–42]. The UPR also promotes inflammation by recruiting Serine/threonine-protein kinase/endoribonuclease IRE1a, which leads to TRAF2-dependent activation of NOD1 and NOD2 and NF-κB activation [38]. This pathway links ER stress to NF-κB-driven inflammation, indicating not only a role for NOD1 and NOD2 in the intracellular surveillance, but also, allowing these receptors to respond to pathogens that do not produce specific PGNs [43].

Based on the central role of NOD1 and NOD2 in these cellular surveillance pathways, these receptors have been proposed as tentative targets for immunomodulation of cancer. In fact, *NOD1* and *NOD2* have already been associated to increased risk of breast cancer [13, 14]. Also, NOD1 activation was shown to promote apoptosis and reduce estrogen-induced proliferative responses in the estrogen-dependent MCF7 breast cancer cell line [44]. Moreover, knockout of *NOD1* in MCF7 cells leads to estrogen-dependent tumor growth in immune deficient mice [45], while its overexpression inhibits estrogen-dependent tumor proliferation in this model. Thus, it has been proposed that *NOD1* may act as a tumor suppressor gene in ER-positive breast cancer cells [44, 45]. Furthermore, it has been previously shown that *NOD1* and *NOD2* have distinct expression patterns among different ER-positive and ER-negative breast cancer cells [46]. To determine whether NOD1 and/or NOD2 play a similar tumor suppressor role in an ER-negative breast cancer cell, we decided to overexpress these receptors in the highly invasive TNBC-derived Hs578T cell line in order to evaluate their impact in breast tumorigenesis in vitro. Overexpression of either *NOD1* or *NOD2* reduces Hs578T cells proliferation and increases their clonogenic potential, suggesting that these receptors may affect tumorigenesis and invasion through ER-independent pathways in this TNBC model. Further investigation of the pathways underlying this phenotype is invaluable to direct future immunomodulatory therapies, especially given their high immunogenicity [11] and the lack of target-directed treatments for TNBCs. Therefore, in the present work, we have performed label-free LC-MS/MS proteome analyses of the NOD1- and NOD2-overexpressing Hs578T cells, integrating the differentially regulated proteins into functional networks to better understand their biological significance in the context of breast cancer progression.

## Results

### Label-free proteomic analysis of Hs578T cell populations

**Fig. 1:**
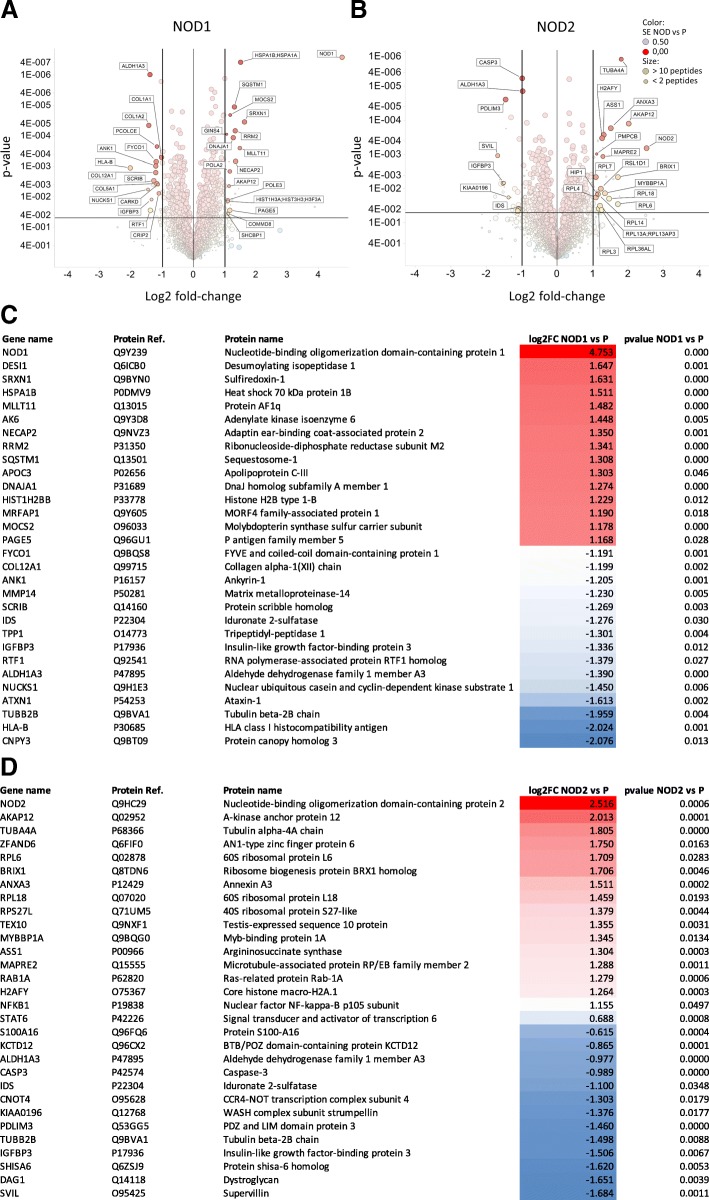
Volcano plots showing detected peptides (represented by annotated Entrez gene names) in samples overexpressing NOD1 (HS578T/NOD1) **(a)** and NOD2 (HS578T/NOD2) **(b)**. Visualization in Spotfire® (TIBCO® Software). Thresholds for differentially expressed gene inclusion were established at + 1 or − 1 log_2_ fold-change (x axis), from the unmodified HS578T cells (P). Similarly, a threshold for inclusion was set at *p*-value 0.05 (y axis). Circles representing each identified protein are colored according to Standard Error (SE) calculated by MSstats and circle size according to the number of peptides identified in each protein. Lists of differentially regulated proteins in HS578T/NOD1 **(c)** and HS578T/NOD2 **(d)** cell populations. Inclusion criteria: log_2_ fold-change ≥ + 1 or ≤ − 1 and p-value ≤0.05. Proteins with log_2_ fold-change ≥ + 0.5 or ≤ − 0.5 and p-value ≤0.01 were also included in the lists. Proteins are ranked and color-coded according to their log_2_-fold-change relative to their expression in the unmodified HS578T cells (P). For each protein, Entrez gene name, Uniprot accession number, protein name and fold change in both experimental groups are reported. Top 30 differentially expressed proteins are shown, complete lists are available as Additional file 1: Figure S1. Color-coding carried out using MS Office Excel, Red: upregulated. Blue: downregulated

**Fig. 2:**
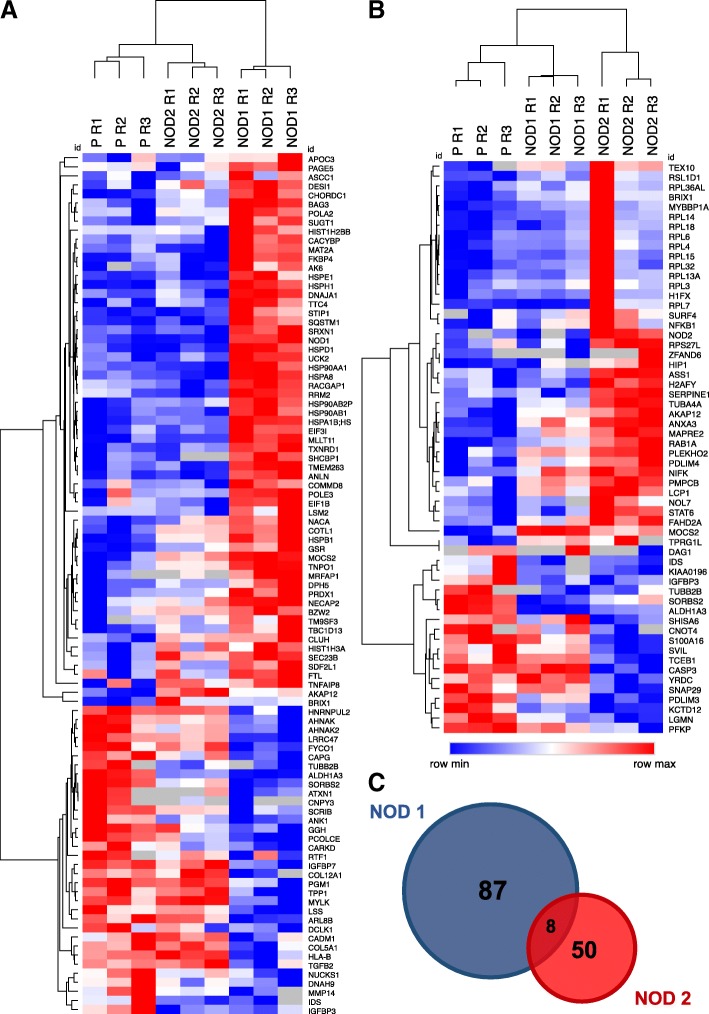
Heatmaps showing clustering of differentially expressed proteins found in NOD1 (HS578T/NOD1) **(a)** and NOD2 (HS578T/NOD2) **(b)** experimental groups. Pearson clustering for rows and columns was carried out according to the log_2_-transformed intensity values (calculated by MaxQuant software) for each of three replicates (R) of unmodified HS578T cells (P), HS578T/NOD1 (NOD1) and HS578T/NOD2 (NOD2) groups in MORPHEUS (Versatile matrix visualization and analysis software; https://software.broadinstitute.org/morpheus) [47]. Rows are Identified by Entrez gene names. Intensities are shown by a color range, from red (row max) to white (row average) and blue (row minimum). **c** Venn diagram showing the distribution of differentially regulated proteins found in NOD1 (HS578T/NOD1; Blue) and NOD2 (HS578T/NOD2; Red) groups, generated in Venny 2.1 (http://bioinfogp.cnb.csic.es/tools/venny) [137]. Circles in scale to group size

In the present study, we examined the effects of *NOD1* and *NOD2* overexpression towards the global proteome of breast cancer-derived Hs578T cells. In our previous work [46], we generated three Hs578T cell subpopulations, via lentiviral transduction of constructs containing either *GFP* alone (HS578T/GFP), or *NOD1* (HS578T/NOD1) or *NOD2* (HS578T/NOD2), both which also express *GFP*. Overexpression of either NOD1 or NOD2 receptors reduces cell proliferation but increases the clonogenic potential in vitro [46]. Elucidating the underlying pathways linking NOD1/NOD2 to tumorigenesis in these cells may reveal new targets for the highly challenging therapy for this highly invasive TNBC model. Therefore, three replicates of each of these cell populations, as well as the unmodified Hs578T cell line (P), were subjected to LC-MS/MS proteomic analysis. A total of 3189 unique proteins were identified in at least two of the three replicates in all four experimental groups. From this complete list, we have found 24 proteins to be downregulated (NOD1 vs P; log_2_ fold-change ≤ − 1, *p*-value ≤0.05), while 31 were upregulated (NOD1 vs P; log_2_ ≥ + 1, *p*-value ≤0.05) in the group overexpressing *NOD1* (HS578T/NOD1; Fig. 1a). Similarly, nine proteins were downregulated (NOD2 vs P; log_2_ fold-change ≤ − 1, p-value ≤0.05) and 33 were upregulated (NOD2 vs P; log_2_ ≥ + 1, p-value ≤0.05) in the HS578T/NOD2 group (Fig. 1b). A second threshold was established to include proteins with high statistical significance (p-value ≤0.01) but lower fold-change (log_2_ fold-change ≥0.5), which added 40 and 16 differentially regulated proteins to the HS578T/NOD1 and HS578T/NOD2 groups, respectively. Proteins with high effect size (log_2_ fold-change ≥1) between the two control groups (HS578T/GFP vs P) were excluded from the analysis. Combining these inclusion parameters, we narrowed down the differentially regulated proteins in the HS578T/NOD1 group to 95 (Fig. 1c), and the HS578T/NOD2 to 58 proteins (Fig. 1d). The top 30 upregulated or downregulated proteins for each experimental group are shown in Fig. 1 (full lists available as Additional file1: Figure S1), while the distribution of these proteins between the two experimental groups is represented in Fig. 2c. Interestingly, the groups of upregulated and downregulated proteins from HS578T/NOD1 and HS578T/NOD2 were considerably dissimilar. Only eight proteins were shared between these two groups (Fig. 2c), while 87 and 50 proteins were exclusively present in the *NOD1-* and *NOD2*-overexpressing groups, respectively. The shared proteins were concordantly up- (MOC2A, AKAP1 and BRX11) or downregulated (SRBS1, IDS, IGFBP-3, AL1A3 and TBB2B) between the two experimental groups (Fig. 1a-d).

### Functional analysis of differentially regulated proteins

A total of 95 and 58 differentially regulated proteins from, respectively, HS578T/NOD1 and HS578T/NOD2, were subjected to a number of Bioinformatics analyses. To visualize the relationships and relative expression of these proteins, the log_2_-transformed intensity values for each of these three replicates (R) in each experimental group (P, NOD1 and NOD2) were subjected to Pearson’s clustering in MORPHEUS [47]. Independent heatmaps for the HS578T/NOD1 (Fig. 2a) and HS578T/NOD2 (Fig. 2b), display clustering for both proteins (rows) and replicates for each group (columns). In general, both HS578T/NOD1 and HS578T/NOD2 groups exhibited a higher number of upregulated proteins and low concurrence of expression between the experimental groups. Additionally, many proteins sharing the same pathways or molecular functions were clustered in proximity. Also, replicates in all experimental groups clustered together, indicating low variability. The only exception was for a cluster of upregulated ribosome-associated proteins in the HS578T/NOD2 group in replicate 1, which deviated from the other replicates. However, statistical significance was still maintained.

### Subcellular localization

NOD1 and NOD2 are cytosolic immune receptors. Hence, the majority of their protein-protein interactions occur in the cytosol, following activation mediated by extracellular stimuli. The complex signal transduction is usually carried further into the nucleus, modulating the function of transcription factors. Therefore, assessing the subcellular localization of the up- and downregulated proteins in the *NOD1* and *NOD2* overexpressing populations, should allow deciphering the pathways which are being affected intracellularly. Two Bioinformatics tools were applied to investigate the subcellular localization of the 95 and 58 differentially regulated proteins in the HS578T/NOD1 and HS578T/NOD groups.

**Fig. 3:**
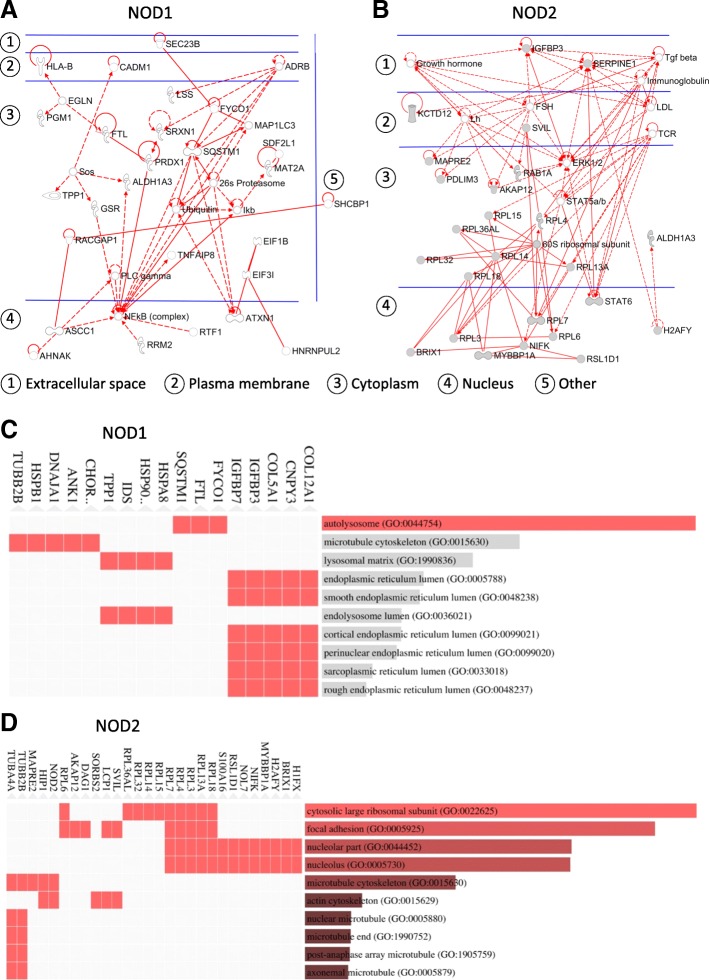
Subcellular localization and cellular component distribution. Subcellular localization and interactions of differentially expressed proteins in groups NOD1 (HS578T/NOD1) **(a)** and NOD2 (HS578T/NOD2) **(b)**, obtained via Qiagen Ingenuity® Pathway Analysis (IPA®). Direct protein interactions are represented by continuous lines, while indirect relationships are represented by dotted lines. Gene Ontology (GO) cellular component term assignment for NOD1 **(c)** and NOD2 **(d)** experimental groups, obtained via EnrichR (http://amp.pharm.mssm.edu/Enrichr) membership analysis (GO database version 2017b). The length of the bar represents the statistical significance of the combined score in Fisher exact test for that specific gene-set or term. In addition, the brighter the color, the more significant that term is. Top supporting protein evidence for each GO cellular component term presented at the left of horizontal bars

Initially, an interaction network analysis with subcellular localization using Ingenuity® Pathway Analysis (IPA®) for the HS578T/NOD1 group, revealed that most of the relevant differentially regulated proteins localize to the cytosol, as expected, with a few notable membrane- (e.g. HLA class I histocompatibility antigen and CADM1) and nuclear-bound (e.g. RIR2 and RTF1) proteins (Fig. 3a). Sequentially, a weighted enrichment analysis (Gene Ontology cellular component term assignment membership analysis) using EnrichR [48], indicated association (Fisher exact test) of these proteins to cytosolic structures, such as *autolysosomes* (e.g. FYCO1, FRIL and SQSTM), *microtubule cytoskeleton* (e.g. ANK1, DNJA1 and TBB2B) and the *lysosomal matrix* (e.g. HSP7C, IDS and TPP1) (Fig. 3c).

Despite having most members localizing to the cytosol, the group of differentially regulated proteins in HS578T/NOD2 presented a higher number of membrane (e.g. SVIL and KCD12), nuclear (e.g. STAT6 and MBB1A) and even extracellularly (e.g. IGFBP-3 and PAI1) localized proteins in the network analysis (IPA®) (Fig. 3b), when compared to HS578T/NOD1 (Fig. 3a). This weighted analysis (EnrichR) supported association of these proteins to cellular structures in the cytosol (*cytosolic large ribosomal subunit*), membrane (*focal adhesion)* and nuclear (*nucleolus*) compartments (Fig. 3d).

### Protein interaction networks

**Fig. 4:**
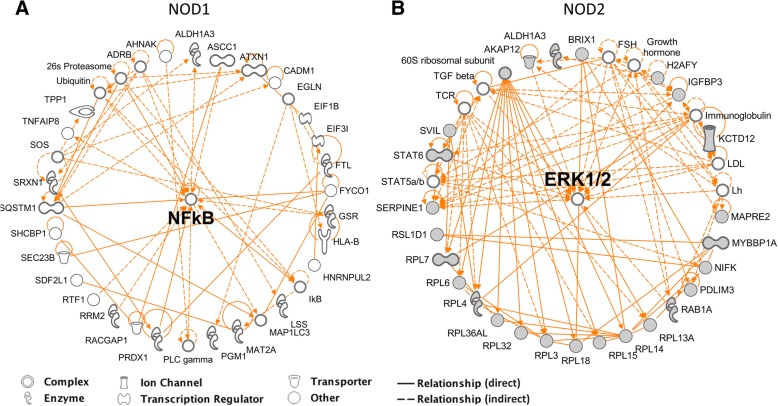
Networks representing protein-protein interactions in differentially regulated proteins from the NOD1 (HS578T/NOD1) **(a)** and NOD2 (HS578T/NOD2) **(b)** experimental groups. Networks generated via Qiagen Ingenuity® Pathway Analysis (IPA®) and organized in radial distribution to emphasize major regulatory nodes. Direct protein interactions are represented by continuous lines, while indirect relationships are represented by dotted lines

To visualize the interactions among proteins identified as differentially regulated, we built radially-distributed interaction networks using IPA®. In agreement with the subcellular network for the proteins in HS578T/NOD1, this analysis indicated that the major pathway affected by *NOD1* overexpression involved NF-kB signaling (Fig. 4a). The centrally located NF-kB complex is directly related to transcription regulators, such as SQSTM and the autophagosome carrier FYCO1. Moreover, protein interactions in HS5778T/NOD2 indicated a major relationship to the ERK1/2 pathway (Fig. 4b), directly interacting with BRX11, IGFBP-3 and RL18.

### Pathway enrichment analysis

**Fig. 5:**
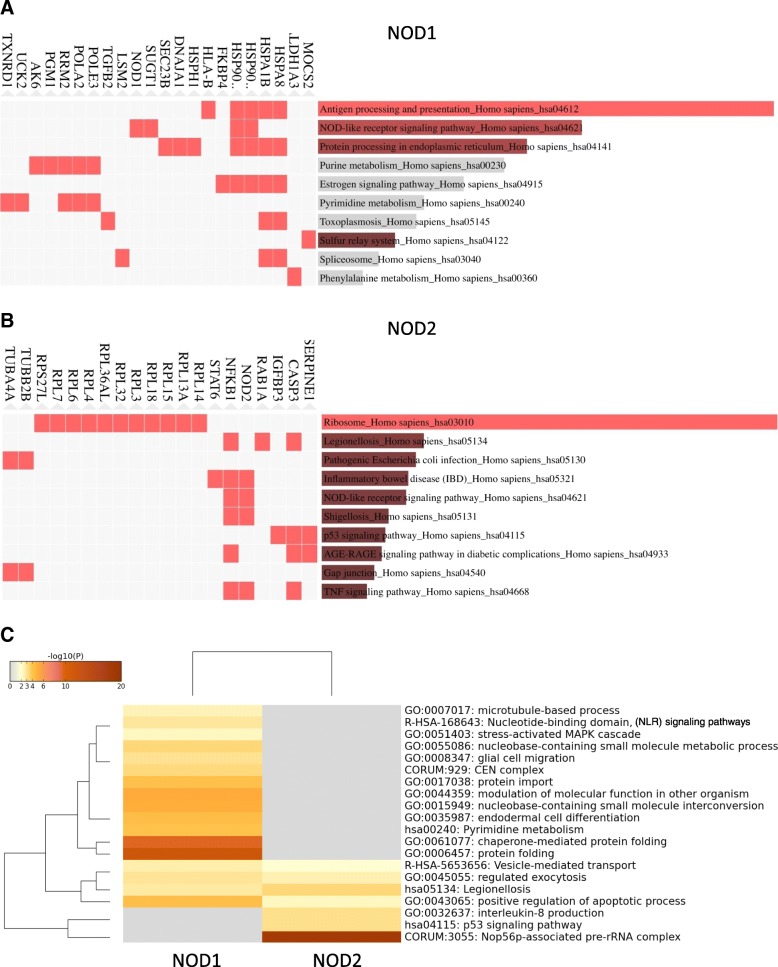
Gene Ontology (GO) pathway enrichment analysis for identified differentially expressed proteins in NOD1 (HS578T/NOD1) **(a)** and NOD2 (HS578T/NOD2) **(b)** groups, generated by EnrichR (http://amp.pharm.mssm.edu/Enrichr) membership analysis, using the KEGG 2016 database. The length of the bar represents the statistical significance of the combined score in Fisher exact test for that specific gene-set or term. In addition, the brighter the color, the more significant that term is. Top supporting protein evidence for each GO cellular component term is presented at the left of horizontal bars. Heatmap **(c)** of GO enriched terms for identified differentially expressed proteins in HS578T/NOD1 and HS578T/NOD2 groups, generated in Metascape (http://metascape.org/gp), using KEGG, Reactome and GO databases. GO terms in heatmap are color coded from grey to brown according to log_10_(p) of the standard accumulative hypergeometric statistical test

To investigate the molecular pathways affected by the overexpression of either *NOD1* or *NOD2*, we subjected the differentially regulated proteins from both HS578T/NOD1 and HS578T/NOD2 groups to pathway enrichment analysis. EnrichR membership analysis using GO terms retrieved from the KEGG 2016 database, indicated that the 95 proteins from HS578T/NOD1 were largely associated to immune-related pathways, such as *Antigen processing and presentation* and *NOD-like receptor signaling*, due to the presence of heat-shock, HLA class I proteins and the SGT1 (Fig. 5a). Notably, there was significant enrichment of nucleotide metabolism pathways (supported by proteins such as DPOA2 and RIR2) and Estrogen signaling (Heat-shock proteins and FKBP4). For the 58 proteins in HS578T/NOD2, the most relevant GO term association was to ribosome-related pathways, supported by the strong presence of RPL proteins, such as RL6 and RL18 (Fig. 5b). Several pathways associated to immune response and inflammation were also enriched, due to the presence of NFKB1, PAI1 and STAT6, among others. A second, unweighted, enrichment analysis was performed in METASCAPE, using KEGG, Reactome and GO databases. A parallel analysis of both experimental groups (Fig. 5c) presented several HS578T/NOD1-exclusive enriched terms (e.g. *microtubule-based processes*, *NLR signaling pathways* and *MAPK cascade*), fewer HS578T/NOD2-exclusive enriched GO terms (e.g. *p53 signaling pathway* and *inrleukin-8 production*) and also GO terms enriched in both groups (e.g. *positive regulation of apoptosis* and *exocytosis*).

### Gene set enrichment analysis (GSEA)

To further explore our proteomic data, an independent analysis was carried out using the GSEA software (Gene Set Enrichment Analysis) [49, 50]. GSEA offers the advantage of a weighted analysis, based on raw MS/MS linear intensity values of all replicates, considering the entire dataset to determine whether molecular signatures (gene sets) show statistically significant differences between two experimental groups. Thus, GSEA may provide an unbiased analysis by our previous inclusion thresholds and statistical testing, which could be compared against our prior results. For the purposes of this analysis, we independently queried the Hallmarks, Reactome and KEGG databases, using the recommended 25% FDR (False Discovery Rate) as a cutoff (Additional file 2: Figure S2).

### HS578T/NOD1

GSEA revealed enrichment of several pathways in HS578T/NOD1, associated to major cellular processes, such as immune response cell cycle, cellular stress, proliferation and cell adhesion. Upregulated pathways with the highest normalized enrichment scores (NES; Additional file 2: Figure S2) were linked to proliferation, such as *nucleotide metabolism* (supported by proteins such as RIR2, UCK2 and DCK), *G2/M checkpoint regulation* (DPOA2, UCK2 and CKS1) and *targets of E2F transcription factors* (RIR2, SLD5 and RGAP1). Additionally, GSEA revealed enrichment of the *DNA replication* pathway, which includes several upregulated members of the MCM (mini-chromosome maintenance proteins) family of proteins (e.g. MCM2, MCM7 and MCM5), which are essential for initiation of eukaryotic genome replication and of the major tumor progression and cellular pathway regulated by the MYC proto-oncogene (e.g. IPO4, MPP10 and MRT4).

Several stress-response pathways were also enriched in HS578T/NOD1. Upregulated proteins were associated to pathways such as: *Reactive oxygen species* (e.g. SRXN1, FRIL, SODC and TRXR1), *Ultraviolet radiation response* (e.g. SQSTM, DPOE3 and DNJA1) and *mTORC1 signaling* (e.g. AF1Q, SQSTM, SDF2L) pathways.

Immune response pathways were also enriched in the *NOD1* overexpressing cells, with proteins associated to *NOD-like receptor signaling* (e.g. SGT1, HS90A and NFKB1) and *antigen processing and ubiquitination* (e.g. UBE2C and UB2E2) pathways. Furthermore, as stress and inflammation signals often induce programmed cell death, Caspase-mediated apoptosis was also enriched in HS578T/NOD1, supported by upregulation of CASP3, CASP4 and SODC.

Downregulated proteins in HS578T/NOD1 were also associated to immune and stress response, as well as to cellular adhesion and migration. Immune related proteins were associated to pathways such as: *Interferon alpha response* (e.g. RIPK2, B2MG and STAT2) and the *natural killer cell cytotoxicity pathway* (e.g. HLA class I histocompatibility antigens 1A02 and 1B51). Downregulated proteins were also associated to stress-related pathways such as: *hypoxia* (e.g. P4HA1, PFKAL and DPYL4) and *ultraviolet response* (CO1A2, RBPMS and ATX10) response systems.

However, pathways with the highest NES were associated to cellular adhesion and migration, including *NCAM signaling proteins* (e.g. CO1A1, CO5A1 and SPTN1), *extracellular matrix organization* (e.g. CO1A2 and MMP14) and *focal adhesion* (KPCA, CO1A1 and MYLK).

### HS578T/NOD2

The gene set enrichment analysis for HS578T/NOD2 revealed mostly relevant upregulated pathways, linked to translation, immune response and tumor progression pathways. Upregulation of several RPL and RPS family members supported the enrichment of the *ribosome* pathway with the highest NES. Upregulation of additional proteins indicated enrichment of other pathways associated to translational regulation, including: *Nonsense mediated decay* (e.g. NCBP1, RS30 and IF4G1) and *3` UTR translational regulation* (e.g. IF2B and IF4A2).

Moreover, enrichment of the *inflammatory response* pathway was supported by upregulation of proteins such as: PAI1 and NFKB1. Finally, similarly to HS578T/NOD1, the pathway regulated by the MYC proto-oncogene was enriched in cells overexpressing *NOD2*, supported by upregulation of proteins such as: FAKD4 and GNL3.

## Discussion

**Fig. 6:**
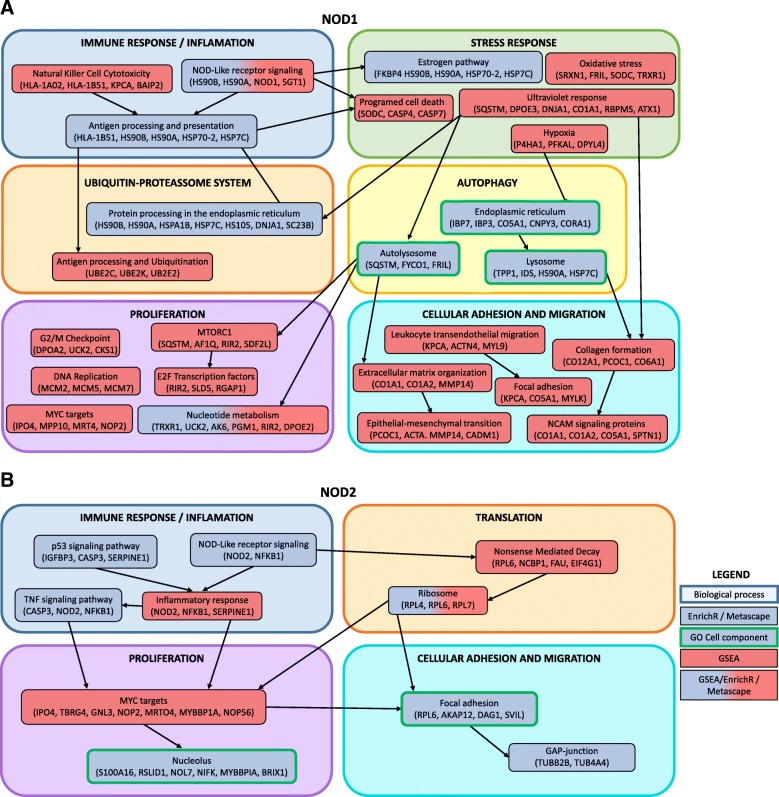
Molecular pathways enriched in HS578T/NOD1 and HS578T/NOD2 according to both EnrichR/Metascape (Blue boxes) and GSEA (Red boxes) Bioinformatics analyses, with examples of upregulated or downregulated proteins supporting each enriched pathway. Red and blue filled boxes represent pathways detected by all methodologies, while green-circled boxes represent cellular component distribution molecular signatures. Pathways are organized under major cellular processes and linked by putative pathway interaction crosstalks

Several immune-related pathways have been associated to tumorigenesis. We have previously shown that overexpression of either NOD1 and NOD2 innate immune receptors impacts cell proliferation and clonogenic potential of the triple negative, breast cancer-derived Hs578T cell line [46]. To further investigate the signaling pathways driving this phenotype, we undertook global proteomic profiling of both *NOD1* and *NOD2* overexpressing cells. The findings from both Bioinformatics enrichment methodologies applied are presented in Fig. 6.

### Alterations in HS578T/NOD1 proteome

#### Immune-related pathways

As expected, overexpression of *NOD1* disrupted signaling pathways related to immune response and inflammation, some of which have been implicated in a variety of cancers [51–54]. Bioinformatics enrichment analysis revealed upregulation of proteins in the *NOD-like receptor signaling pathway*, including several heat shock proteins and SGT1, which in turn interacts with HSP90 [55] and is essential for NOD1-mediated cytokine production and apoptosis in breast cancer cells [56]. Moreover, SGT1 is involved in kinetochore formation, being required for the G1/S and G2/M transitions [57–59], directly linking NOD-like signaling to cell proliferation control. Several upregulated heat shock proteins were identified as being part of the *antigen processing and presentation* pathway alongside 1B51 (*HLA-B* gene), a downregulated membrane-bound MHC class I molecule which plays a central role in the immune system by presenting peptides derived from the endoplasmic reticulum lumen [60]. Upon activation, *HLA-B* can signal to the NF-κB complex via FRIL and PRDX1 (Fig. 3a) [61]. NF-κB signaling is purportedly one of the most disrupted pathways in this model, as supported by the number of interactions in IPA radial network (Fig. 4a). The co-chaperone FKBP4, an HSP90 interactor, was also upregulated in HS578T/NOD1. FKBP4 participates in estrogen signaling by trafficking steroid hormone receptors between cytoplasmic and nuclear compartments [62] and regulates microtubule dynamics by inhibiting MAPT/TAU [63–66].

#### Stress-related pathways

The immune-related pathways enriched in HS578T/NOD1, which included several heat shock proteins, are closely related to stress response systems. Accordingly, a considerable number of stress associated pathways were found to be enriched in HS578T/NOD1, such as the *Programmed cell death* pathway, which includes a few Caspase molecules, and the *Oxidative stress pathway*, which includes SRXN1, SODC and FRIL, crosstalking with the HLA signaling pathway. Other apoptosis-associated proteins, such as TFIP8, a tumor suppressor that regulates TNF-mediated apoptosis via inhibition of caspase-8 [67] were also upregulated in HS578T/NOD1. Molecular pathways associated to hypoxia and UV radiation stress response were also disrupted in the *NOD1* overexpressing cells, as indicated by upregulation of key regulatory proteins such as SQSTM.

#### Protein degradation pathways

Hypoxia and UV radiation stress response pathways, which were disrupted in HS578T/NOD1, are known to activate stress-induced autophagy [68, 69]. Constitutive autophagy is an essential housekeeping process to maintain cellular homeostasis by targeting cytosolic components and organelles for degradation in the lysosome. However, autophagy is also highly responsive to stress [70], being activated through several stress response pathways which often employ heat shock, chaperone and co-chaperone proteins from the unfolded protein response (UPR) system. UPR is usually triggered by accumulation of misfolded proteins in the endoplasmic reticulum (ER stress) and, aside from being a potent trigger for autophagy, can also induce apoptosis through inflammatory pathways [68]. NOD1 and NOD2 were shown to be important mediators of this ER stress-induced inflammation, via NF-κB signaling [39]. Also, NOD1 can directly interact with bacterial peptidoglycans in early endosomes to promote autophagy and inflammation, independently of the UPR system [23]. Moreover, both NOD1 and NOD2 can activate autophagy during bacterial infection by a mechanism which is independent of RIP2 and NF-κB, by recruiting the autophagy protein ATG16L1 to the plasma membrane at the bacterial entry site [34, 35].

Our analyses revealed that both the lysosome and autolysosome molecular signatures were enriched in HS578T/NOD1, suggesting stress-related disruption of the autophagy system. A key protein in this system, SQSTM, was upregulated, being associated to both stress response and autophagy enriched pathways. SQSTM is a highly selective cargo receptor protein [71], directly interacting with cytosolic targets for stress-induced autophagy degradation [72–75].

SQSTM is also involved in the formation and autophagic degradation of cytoplasmic ubiquitin-containing inclusions [73, 74]. Enrichment of the *Antigen processing and Ubiquitination* pathway, supported by upregulation of several Ubiquitin-conjugating enzymes (e.g. UBE2C, UBE2K and UB2E2) indicates activation of the Ubiquitin-Proteasome System (UPS) in HS578T/NOD1. Additionally, Ubiquitin was reported to bind both NOD1 and NOD2 to regulate inflammation and autophagy [76]. Furthermore, our protein interaction networks (Figs. 3, 5a) pointed to UPS as one of the major interacting nodes, linking the function of vesicle-trafficking proteins, such as SC23B, SQSTM and FYCO1, to the NFκB signaling pathway. Finally, upregulation of SQSTM, alongside other proteins, such as AF1Q (*MLLT11*), suggests activation of the mTORC1 complex, indicating yet another possible crosstalk between the Ubiquitin-Proteasome and autophagy systems [77].

#### Cellular adhesion and migration pathways

Autophagy has been described as a regulator of cell migration, modulating tumor cell motility, invasion and metastasis [78–80]. Overexpression of autophagy cargo receptors has been associated with the more aggressive mesenchymal subtype of primary glioblastoma, in which SQSTM was required for invasion and migration of stem-like cancer cells [78, 81, 82].

Furthermore, during bacterial invasion, the inflammatory response promoted by NOD1 and NOD2 directly modulates the expression of adhesion molecules, such as E-Cadherin [83], ICAM1 [84] and VCAM1 [85]. Also, stimulation of *NOD1* in epithelial cancer cells promotes specific pro-tumorigenic effects that include modulation of ICAM1 [53].

These data suggest that overexpression of *NOD1* and the enrichment of autophagy-related pathways and proteins such as SQSTM in HS578T/NOD1 may directly lead to modulation of adhesion and extracellular matrix organization pathways. Indeed, several molecular signatures related to migration and adhesion were altered in HS578T/NOD1, including *Focal Adhesion*, *Epithelial-mesenchymal transition, NCAM signaling proteins* and *Extracellular matrix organization*. Modulation of these pathways leading to an increase in migration and invasiveness in breast cancer cells [6], supports the phenotype of increased colony formation potential previously observed in HS578T/NOD1 [46].

#### Cellular proliferation pathways

Upregulation of SQSTM and enrichment of the mTORC1 pathway in HS578T/NOD1 may directly modulate cellular proliferation, via the PI3K/Akt axis [86, 87]. The PI3K/Akt/mTOR signaling pathway is often deregulated in cancers, modulating cell growth, apoptosis, malignant transformation, tumor progression and metastasis [6, 88, 89]. One of the proteins in the PI3K/Akt pathway, SCRIB, was downregulated in HS578T/NOD1. This tumor suppressor coordinates cell proliferation by regulating progression from G1 to S phase [90] and has also been reported as a positive regulator of apoptosis during acinar morphogenesis of the mammary epithelium [91]. Furthermore, SCRIB has a role in cell migration and adhesion, regulating cell invasion through MAPK signaling [92], also indicated by enrichment of the *Stress-activated MAPK cascade* pathway in Metascape.

Other members of the mTORC1 complex may also impact *NOD1*-overxpressing cells proliferation, as, for example, RIR2, a pyrimidine catalytic subunit, which is present in the *E2F transcription factor network*. Upregulation of RIR2 in HS578T/NOD1 may also repress proliferation through inhibition of Wnt [93]. The Wnt pathway may be further repressed in these cells by downregulation of RTF1, a component of the PAF1 complex, required for transcription of Wnt target genes [94, 95]. Also, the nuclear cyclin-dependent kinase NUCKS was found to be downregulated in HS578T/NOD1. This cell-cycle protein was implicated in tumorigenesis, progression and poor prognosis of several human malignancies [96].

The cross-talk between these cell cycle-related pathways, as well as others, such as *G2/M checkpoint, MYC targets* and *DNA replication*, found to be enriched in our analysis, point to several avenues through which overexpression of NOD1 may modulate cell proliferation. Dysregulation of these pathways might be the underlying mechanism of the reduced proliferation phenotype observed in HS578T/NOD1 cells [46].

### Alterations in HS578T/NOD2 proteome

#### Immune-related pathways

Bioinformatics analyses of the proteome of *NOD2*-overexpressing cells also suggested an upregulation of pathways involved in immune response and inflammation. Enrichment of pathways, such as the *NOD-Like receptor signaling*, *Inflammation response* and *TNF signaling pathways*, were supported by dysregulation of proteins, such as: CASP3, NFKB1 and the serine proteinase inhibitor PAI1 (*SERPINE1*). PAI1, detected in the *inflammatory response pathway* alongside NOD2, was reported to activate peptidoglycan-induced inflammation and autophagy in rat macrophages [97, 98], further linking the NOD inflammatory signaling to protein degradation pathways.

Furthermore, downregulation of IGFBP-3 and upregulation of PAI1 suggest the involvement of the *p53 signaling pathway*. IGFBP-3, a direct p53 effector, has been shown to modulate proliferation by altering the interaction of IGFs to their cell surface receptors [99, 100]. Other proteins in the p53 pathway, such as the pro-tumorigenic GNL3, also supported the enrichment of *MYC targets* molecular signature. Targets of the MYC proto-oncogene are frequently observed in several human cancers, modulating cell cycle progression, apoptosis and cellular transformation [101].

PAI1 and IGFBP-3 are also associated to the TGF-beta receptor signaling pathway [102], an interaction which was indicated in our HS578T/NOD2 protein network (Fig.3 b), as well as their downstream signaling to the major interacting node ERK1/2, (Fig. 4b).

#### Cellular proliferation

Involvement of ERK1/2 suggests that proliferation and survival may be regulated by the MAPK signaling pathway [103–105]. Activation of ERK1/2 generally promotes proliferation and cell survival, however, under certain conditions, ERK1/2 may have pro-apoptotic functions, either by translocating to the nucleus [106, 107], or signaling through a cascade involving STAT proteins. Specifically, ERK1/2 can activate STAT6, which was upregulated in HS578T/NOD2. Other targets of ERK1/2 signaling are also upregulated in the NOD2 overexpressing cells, such as nucleolar MK67I (*NFIK*), which regulates mitosis by interacting with the Ki-67 antigen, [108] and the tumor suppressor and cell cycle regulator NOL7 [109].

One of the outcomes of ERK1/2 signaling modulation is the dysregulation of protein synthesis pathways, as supported by the differential expression of several RPL and RPS proteins in HS578T/NOD2. Upregulation of several of these RPL proteins may be overestimated due to their higher expression in NOD2 replicate 1 (Fig. 2b). However, significance was maintained across statistical tests in both independent enrichment approaches. Bona fide upregulation of these proteins was also supported by the differential expression of correlate proteins, such as the eukaryotic translation initiation IF4G1, part of the complex responsible for mRNA loading to the ribosome [110].

#### Cellular adhesion and migration pathways

Dysregulation of ERK1/2 signaling may also be responsible for altering adhesion and migration of the *NOD2*-overexpressing cells. Several differentially regulated proteins in HS578T/NOD2 were identified in the *Focal adhesion* pathway, such as: the anchoring protein AKAP1, also shown to support mTOR-dependent tumor growth in breast cancer cells [111]; the actin-binding protein Plastin-2 (LCP-1) [112]; the actin-filament membrane anchor SVIL, which plays a critical role in tumor invasion [113]; and DAG1, a central component of dystrophin-glycoprotein complex that links the extracellular matrix to the cytoskeleton [114].

Furthermore, upregulation of PAI1 in HS578T/NOD2 is a direct evidence of cell adhesion modulation. As PLAU inhibitor, PAI1 is directly involved in extracellular matrix remodeling and cell adhesion [115], promoting tumor progression and metastasis in several cancers, including breast cancer [116]. Interestingly, the effects of PAI1 in breast tumor progression seems to be regulated by a non-canonical TGF-beta1 pathway [102, 117, 118]. Moreover, it has also been proposed that PAI1 may regulate cell migration independently of its role as protease inhibitor [119, 120]. Modulation of these proteins may partially explain the in vitro phenotype of increased colony formation potential observed in the *NOD2*-overexpressing cells [46] .

Our bioinformatics analyses on HS578T/NOD2 are consistent with a previous proteome analysis of HEK293 cells overexpressing NOD2, in which most of the differentially regulated proteins were associated to: biosynthesis, modification, or degradation of proteins; heat shock or protein folding; and DNA repair and replication [121]. Our Bioinformatics analyses on HS578T/NOD2 are consistent with a previous proteome analysis of HEK293 cells overexpressing NOD2, in which most of the differentially regulated proteins were associated to: biosynthesis, modification, or degradation of proteins; heat shock or protein folding; and DNA repair and replication [121].

#### Shared proteome alterations in HS578T/NOD1 and HS578T/NOD2 cells

Despite their sequence similarities, NOD1 and NOD2 recognize different molecular patterns, being able to activate specific downstream pathways [38, 122], as reflected in the distribution of differentially regulated proteins between HS578T/NOD1 and HS578T/NOD2 (Fig. 2c). Despite the small protein overlap, the two groups shared several disrupted signaling pathways (Fig. 6), some of which are represented in the eight differentially regulated proteins shared between the two groups. These proteins include: a) A component of the translation machinery (BRX11) b) An IGF-binding protein (IGFBP-3) associated to TGF-beta and ERK1/2 (Fig. 3b), shown to modulate proliferation and apoptosis in vitro [99, 100]. c) Iduronate 2-sulfatase, which may indicate dysregulation of lysosomal degradation. Also, three cytoskeleton-associated proteins, namely: d) TBB2B, which is a major component of microtubules [123], e) AKAP1, shown to support mTOR-dependent tumor growth in breast cancer cells [111] and f) SRBS1, an adapter protein which regulates the assembly of kinase signaling complexes bound to the actin cytoskeleton. These signaling complexes promote protein interactions which induce both ubiquitination/degradation [124] and phosphorylation of targets [125]. One of these interactions is the AKT1-mediated activation of PAK1, indicating a critical role for SRBS1 in the PI3K/Akt/mTOR signaling pathway, as indicated by SRBS1-dependent signaling regulation of pancreatic cell migration, adhesion and tumorigenicity [126].

## Conclusions

In this work, we applied high-throughput LC-MS/MS analyses to characterize the proteome of the Hs578T triple negative breast cancer-derived cell line, overexpressing either NOD1 or NOD2 receptors. We have previously reported that these cells have reduced proliferation and increased clonogenic potential in vitro [46]. The proteomic analysis suggests that overexpression of both *NOD1* and *NOD2* disrupt immune related pathways, notably those signaling through NF-κB. The NF-κB complex displayed a central role in our interaction networks, seemingly activated via antigen presentation signals (HLA class I histocompatibility proteins) and TNF signaling pathways. In the *NOD1* overexpressing cells, upregulation of these immune-related pathways seems to modulate several stress response systems. In turn, immune and stress pathways may modulate both proteasome and autophagy degradation systems, and ultimately, dysregulate both proliferation and cellular adhesion/migration via PI3K/Akt/mTOR and MAPK signaling pathways. In *NOD2*-overexpressing cells upregulation of immune-related pathways also seems to disrupt proliferation through MAPK signaling, via modulation of TNF and p53 pathways.

Further investigation of these pathways and their crosstalk in this breast cancer model may provide insights into relevant targets for therapeutic intervention, possibly enabling immunomodulation of tumorigenesis in aggressive triple negative breast cancers.

## Methods

### Cell models

Development and culture conditions for parental (unmodified) or transduced triple-negative (ER-/PR-/HER2-) Hs578T (ATCC® HTB-126™) cells were performed as previously described [46]. Transduced cells overexpress either GFP alone (HS578T/GFP), *NOD1* (HS578T/NOD1) or *NOD2* (HS578T/NOD2) genes. Of note, both HS578T/NOD1 and HS578T/NOD2 transduced cells co-express GFP (mediated by an IRES sequence downstream of respective NOD1/2 cDNAs into the original vectors).

### Sample preparation for proteomic analysis

Cells from each experimental group were cultured independently, in triplicates, to obtain total lysate samples. For each sample, 2 × 10^6^ cells were harvested by trypsin digestion and proteins were extracted in 8 M urea, 50 mM ammonium bicarbonate buffer, and subsequently digested with trypsin. Briefly, cysteine disulfide bonds were reduced with 5 mM Tris(2-carboxyethyl) phosphine (TCEP) at 30 °C for 60 min, followed by cysteine alkylation with 15 mM iodoacetamide (IAA) in the dark, at room temperature for 30 min. Following alkylation, urea was diluted to 1 M urea using 50 mM ammonium bicarbonate, and proteins were finally subjected to overnight digestion with mass spec grade Trypsin/Lys-C mix (Promega, Madison, WI). The digested proteins were desalted using AssayMap C_18_ cartridges mounted on a BRAVO liquid handling system (Agilent, Columbia, MD), and the organic solvent was removed in a SpeedVac concentrator prior to LC-MS/MS analysis.

### LC-MS/MS procedures

Dried samples were reconstituted with 2% acetonitrile, 0.1% formic acid and analyzed by LC-MS/MS using a Proxeon EASY nanoLC system (Thermo Fisher Scientific) coupled to an Orbitrap Fusion Lumos mass spectrometer (Thermo Fisher Scientific). Peptides were separated using an analytical C_18_ Acclaim PepMap column 0.075 × 500 mm, 2 μm particles (Thermo Scientific) in a 90-min linear gradient of 2–28% solvent B at a flow rate of 300 nL/min. The mass spectrometer was operated in positive data-dependent acquisition mode. MS1 spectra were measured with a resolution of 120,000, an AGC target of 1e6, a maximum injection time of 100 ms and a mass range from 350 to 1400 m/z. The instrument was set to run at top speed mode with 3 s cycles for the survey and the MS/MS scans. After a survey scan, tandem MS was performed on the most abundant precursors exhibiting a charge state from 2 to 8 of greater than 5e3 intensity by isolating them in the quadrupole at 0.8 Th. HCD fragmentation was applied with 30% collision energy and the resulting fragments were detected using the turbo scan rate of the ion trap. The AGC target for MS/MS was set to 1e4 and the maximum injection time limited to 15 ms. The dynamic exclusion was set to 15 s with a 10 ppm mass tolerance around the precursor and its isotopes.

### LC-MS/MS data analysis

All mass spectra were analyzed with MaxQuant software version 1.5.5.1 [127]. MS/MS spectra were searched against the *Homo sapiens* Uniprot protein sequence database (version July 2017) and GPM cRAP sequences (commonly known protein contaminants). Precursor mass tolerance was set to 20 ppm and 4.5 ppm for the first search where initial mass recalibration was completed and for the main search, respectively. Product ions were searched with a mass tolerance 0.5 Da. The maximum precursor ion charge state used for searching was 7. Carbamidomethylation of cysteines was searched as a fixed modification, while oxidation of methionines and acetylation of protein N-terminal were searched as variable modifications. Enzyme was set to trypsin in a specific mode and a maximum of two missed cleavages was allowed for searching. The target-decoy-based false discovery rate (FDR) filter for spectrum and protein identification was set to 1%.

The mass spectrometry data have been deposited in the ProteomeXchange Consortium [128] via the PRIDE partner repository with the dataset identifier PXD012542.

### Statistical analysis

The evidence table output from MaxQuant was used for label-free protein quantitative analysis. First, calculated peptide intensities were log_2_-transformed and normalized across samples to account for systematic errors. A total of 8 normalization approaches were deployed (*Loess, Robust Linear Regression, Variance Stabilization and Normalization, Total Intensity, Median Intensity, Average Intensity, NormFinder* and *Quantile*), and their performance assessed [129] in order to determine the optimal normalization method (herein Loess normalization). Following normalization, all non-razor peptide sequences were removed from the list. Protein-level quantification and testing for differential abundance were performed using MSstats bioconductor package [130, 131] based on a linear mixed-effects model. The model decomposes log-intensities into the effects of technical and biological replicates, peptides and statistical interactions.

### Bioinformatic analysis

The initial list was filtered to remove protein contaminants and proteins not detected in at least two of three replicates from each experimental group. Differentially regulated proteins were selected from the HS578T/NOD1 and HS578T/NOD2 experimental groups according to two inclusion thresholds, namely: (1) ≥ + 1 or ≤ − 1 log_2_ fold-change from the unmodified Hs578T cells (P) and *p*-value of ≤0.05. (2) ≥ + 0.5 or ≤ − 0.5 log_2_ fold-change from the unmodified Hs578T cells (P) and p-value ≤0.01. Additionally, proteins with log_2_ fold-change ≥ + 1 or ≤ − 1 between the two control groups (HS578T/GFP vs P) were excluded.

Protein interaction networks, both organized by subcellular localization and by major interaction node (radial distribution), were obtained using the Ingenuity IPA™ software (Ingenuity Systems, QIAGEN) from the lists of differentially regulated proteins.

Gene Ontology (GO) cellular component term assignment for HS578T/NOD1 and HS578T/NOD2 were obtained via EnrichR [48, 132, 133] membership analysis from the lists of differentially regulated proteins. Bar graph based on GO database version 2017b, in which the bar length represents the statistical significance of the combined score of that specific gene-set or term. Combined score is computed by taking de log of the p-value from the Fisher exact test and multiplying that by the z-score of the deviation of the expected rank. Additionally, the brighter the color, the more significant that term is. Supporting protein evidence, from the differentially expressed lists, for each GO cellular component term is presented at the left of horizontal bars.

Gene Ontology pathway enrichment analysis was also generated independently for both experimental groups, by EnrichR membership analysis, using the KEGG 2016 database. Bar length represents the statistical significance of the combined score (see above). Supporting protein evidence for each GO cellular component term is presented at the left of horizontal bars. Independent pathway enrichment analysis in Metascape [134, 135] was run concomitantly on HS578T/NOD1 and HS578T/NOD2 protein lists. KEGG, Reactome and GO database results are presented as enriched GO term Heatmap, color-coded from grey to brown according to log_10_(p) of the standard cumulative hypergeometric statistical test. Weighted enrichment pathway analysis of the complete set of 3189 identified proteins was performed using GSEA (Gene Set Enrichment Analysis) 3.0 software [49, 50, 136]. Linear intensity values for each protein in the three replicates from Hs578T/NOD1, HS578T/NOD2 and parental control group were used to determine enriched gene sets (1000 permutations) from the following databases: *h.all.v6.1.symbols.gmt [Hallmarks]; c2.cp.kegg.v6.1.symbols.gmt [curated]; c2.cp.reactome.v.6.1.symbols.gmt [curated].*

## Additional files

Additional file 1: Full list of Differentially regulated proteins: Differentially regulated proteins in HS578T/NOD1 (A) and HS578T/NOD2 (B) cells. Proteins are ranked and color coded by their log_2_-fold change relative to unmodified Hs578T cells (P). For each protein, Entrez gene name, Uniprot accession number, protein name and fold-change in both experimental groups are reported. Color coding performed in MS Office Excel, Red: upregulated. Blue: downregulated. (PDF 441 kb)

Additional file 2: GSEA gene sets Enriched gene sets detected by GSEA in HS578T/NOD1 (A) and HS578T/NOD2 (B) cells. Gene sets are ranked and color coded by their NES (Normalized Enrichment Score) relative to unmodified Hs578T cells (P). For each gene set, name and size of the gene set, ES (Enrichment Score), NES, nominal *p*-value and FDR (False Discovery Rate) are reported. Color coding performed in MS Office Excel, Red: upregulated. Blue: downregulated. (PDF 215 kb)

## Abbreviations

1A02: HLA class I histocompatibility antigen, A-2 alpha chain
1B51: HLA class I histocompatibility antigen, B-51 alpha chain
AF1Q: Protein AF1q
AKAP1: A-kinase anchor protein 12
AL1A3: Aldehyde dehydrogenase family 1 member A3
ANK1: Ankyrin-1
AR: Androgen Receptor
ATX10: Ataxin-10
B2MG: Beta-2-microglobulin
BRX11: Ribosome biogenesis protein BRX1 homolog
CADH1: E-Cadherin
CADM1: Cell adhesion molecule 1
CASP3: Caspase-3
CASP4: Caspase-4
CKS1: Cyclin-dependent kinases regulatory subunit 1
CO1A1: Collagen alpha-1(I) chain
CO1A2: Collagen alpha-2(I) chain
CO5A1: Collagen alpha-1(V) chain
CO6A1: Collagen alpha-1(VI) chain
COCA1: Collagen alpha-1(XII) chain
DAG1: Dystroglycan
DAMP: Danger-Associated Molecular Patterns
DCK: Deoxycytidine kinase
DNJA1: DnaJ homolog subfamily A member 1
DPOA2: DNA polymerase alpha subunit B
DPOE3: DNA polymerase epsilon subunit 3
DPYL4: Dihydropyrimidinase-related protein 4
ECAD: E-Cadherin
EIF1B: Eukaryotic translation initiation factor 1b
eIF-4G1: Eukaryotic translation initiation factor 4 gamma 1
ER: Estrogen Receptor
FAKD4: FAST kinase domain-containing protein 4
FDR: False discovery rate
FKBP4: Peptidyl-prolyl cis-trans isomerase FKBP4
FRIL: Ferritin light chain
FTL: Ferritin light chain
FYCO1: FYVE and coiled-coil domain-containing protein 1
GNL3: Guanine nucleotide-binding protein-like 3
GO: Gene Ontology
GSEA: Gene Set Enrichment Analysis
H2B1B: Histone H2B type 1-B
H31: Histone H3.1
HER2: Human Epidermal Growth Factor Receptor 2
HLA-A: HLA class I histocompatibility antigen, A-2 alpha chain
HLA-B: HLA class I histocompatibility antigen, B-51 alpha chain
HS90A: Heat shock protein HSP 90-alpha
HSP-60: 60 kDa heat shock protein, mitochondrial
HSP70–2: Heat shock 70 kDa protein 1B
HSP7C: Heat shock cognate 71 kDa protein
HSP90: Heat-shock protein 90
IAA: Iodoacetamide
ICAM1: Intercellular Adhesion Molecule 1
IDS: Iduronate 2-sulfatase
iE-DAP: Gamma-D-glutamyl-meso-diaminopimelic acid
IF2B: Eukaryotic translation initiation factor 2 subunit 2
IF4A2: Eukaryotic initiation factor 4A-II
IF4G1: Eukaryotic translation initiation factor 4 gamma 1
IGFBP-3: Insulin-like growth factor-binding protein 3
IKK: Inhibitory κB Kinase
IPO4: Importin-4
KCD12: BTB/POZ domain-containing protein KCTD12
KPCA: Protein kinase C alpha type
LCP-1: Plastin-2
LRR: Leucine-Rich Repeat domain
MAPK: Mitogen-Activated Protein Kinase
MBB1A: Myb-binding protein 1A
MCM: Mini-chromosome maintenance proteins
MCM2: DNA replication licensing factor MCM2
MCM5: DNA replication licensing factor MCM5
MCM7: DNA replication licensing factor MCM7
MDP: Muramyl Dipeptide
MK67I: MKI67 FHA domain-interacting nucleolar phosphoprotein
MMP14: Matrix metalloproteinase-14
MOC2A: Molybdopterin synthase sulfur carrier subunit
MPP10: U3 small nucleolar ribonucleoprotein protein MPP10
MRT4: mRNA turnover protein 4 homolog
MYLK: Myosin light chain kinase, smooth muscle
NBR1: Next to BRCA1 gene 1 protein
NCBP1: Nuclear cap-binding protein subunit 1
NES: Normalized enrichment scores
NF-κB: Nuclear factor κB
NFKB1: NF-kappa-B p105 subunit
NGF: Beta-nerve growth factor
NLR: NACHT and Leucine Rich Repeat domain containing protein
NOD1: Nucleotide-Binding Oligomerization Domain-Containing Protein 1
NOD2: Nucleotide-Binding Oligomerization Domain-Containing Protein 2
NOL7: Nucleolar protein 7
NUCKS: Nuclear ubiquitous casein and cyclin-dependent kinase substrate 1
P4HA1: Prolyl 4-hydroxylase subunit alpha-1
PAI1: Plasminogen activator inhibitor 1
PAMP: Pathogen-Associated Molecular Pattern
PCOC1: Procollagen C-endopeptidase enhancer 1
PDLI1: PDZ and LIM domain protein 3
PFKAL: ATP-dependent 6-phosphofructokinase, liver type
PGN: Peptidoglycan
PLAU: Urokinase-type plasminogen activator
PNP: Purine nucleoside phosphorylase
PR: Progesterone Receptor
PRDX1: Peroxiredoxin-1
PRR: Pattern Recognition Receptor
RBPMS: RNA-binding protein with multiple splicing
RGAP1: Rac GTPase-activating protein 1
RIPK2: Receptor-interacting serine/threonine-protein kinase 2
RIR2: Ribonucleoside-diphosphate reductase subunit M2
RIR2B: Ribonucleoside-diphosphate reductase subunit M2 B
RL18: 60S ribosomal protein L18
RL6: 60S ribosomal protein L6
RS30: 40S ribosomal protein S30
RTF1: RNA polymerase-associated protein RTF1 homolog
SC23B: Protein transport protein Sec23B
SCRIB: Protein scribble homolog
SDF2L: Stromal cell-derived factor 2-like protein 1
SGT1: Protein SGT1 homolog
SLD5: DNA replication complex GINS protein SLD5
SODC: Superoxide dismutase [Cu-Zn]
SPTN1: Spectrin alpha chain, non-erythrocytic 1
SQSTM: Sequestosome-1
SRBS1: Sorbin and SH3 domain-containing protein 2
SRBS2: Sorbin and SH3 domain-containing protein 2
SRXN1: Sulfiredoxin-1
STAT2: Signal transducer and activator of transcription 2
STAT6: Signal transducer and activator of transcription 6
SVIL: Supervillin
TAK1: TGF-β-activated kinase 1
TBB2B: Tubulin beta-2B chain
TCEP: Tris(2-carboxyethyl)phosphine
TFIP8: Tumor necrosis factor alpha-induced protein 8
TNBC: Triple Negative Breast Cancer
TPP1: Tripeptidyl-peptidase 1
TRAF: TNF receptor-associated factors
Tri-DAP: L-Ala-y-D-Glu-mDAP
TRXR1: Thioredoxin reductase 1, cytoplasmic
TYSY: Thymidylate synthase
UB2E2: Ubiquitin-conjugating enzyme E2 E2
UBE2C: Ubiquitin-conjugating enzyme E2 C
UBE2K: Ubiquitin-conjugating enzyme E2 K
UCK2: Uridine-cytidine kinase 2
UPR: Unfolded protein response
UPS: Ubiquitin-Proteasome System
VCAM1: Vascular Cell Adhesion Molecule 1
WASF1: Wiskott-Aldrich syndrome protein family member 1
