# Supplementary material for: Proteome profiling of triple negative breast cancer cells overexpressing NOD1 and NOD2 receptors unveils molecular signatures of malignant cell proliferation
Source: BMC Genomics. 2019 Feb 21;20:152. doi: 10.1186/s12864-019-5523-6 (PMC6385390; doi:10.1186/s12864-019-5523-6)
Supplement: Supplementary file 1 — Full list of Differentially regulated proteins: Differentially regulated proteins in HS578T/NOD1 (A) and HS578T/NOD2 (B) cells. Proteins are ranked and color coded by their log2-fold change relative to unmodified Hs578T cells (P). For each protein, Entrez gene name, Uniprot accession number, protein name and fold-change in both experimental groups are reported. Color coding performed in MS Office Excel, Red: upregulated. Blue: downregulated. (PDF 441 kb) [file 12864_2019_5523_MOESM1_ESM.pdf]

A

Gene name	Protein Ref.	Protein name	log2FC NOD1 vs P	pvalue NOD1 vs P	log2FC NOD2 vs P	pvalue NOD2 vs P
NOD1	Q9Y239	Nucleotide-binding oligomerization domain-containing protein 1	4.753	0.000	-0.430	0.1920
DESI1	Q6ICB0	Desumoylating isopeptidase 1	1.647	0.001	0.864	0.0361
SRXN1	Q9BYN0	Sulfiredoxin-1	1.631	0.000	-0.083	0.6848
HSPA1B	P0DMV9	Heat shock 70 kDa protein 1B	1.511	0.000	0.250	0.0383
MLLT11	Q13015	Protein AF1q	1.482	0.000	0.012	0.9610
AK6	Q9Y3D8	Adenylate kinase isoenzyme 6	1.448	0.005	0.067	0.8585
NECAP2	Q9NVZ3	Adaptin ear-binding coat-associated protein 2	1.350	0.001	0.555	0.0586
RRM2	P31350	Ribonucleoside-diphosphate reductase subunit M2	1.341	0.000	-0.540	0.0163
SQSTM1	Q13501	Sequestosome-1	1.308	0.000	0.090	0.5252
APOC3	P02656	Apolipoprotein C-III	1.303	0.046	0.202	0.7234
DNAJA1	P31689	DnaJ homolog subfamily A member 1	1.274	0.000	0.090	0.6346
HIST1H2BB	P33778	Histone H2B type 1-B	1.229	0.012	-0.305	0.4476
MRFAP1	Q9Y605	MORF4 family-associated protein 1	1.190	0.018	0.611	0.2445
MOCS2	O96033	Molybdopterin synthase sulfur carrier subunit	1.178	0.000	0.729	0.0006
PAGE5	Q96GU1	P antigen family member 5	1.168	0.028	-0.483	0.2985
POLA2	Q14181	DNA polymerase alpha subunit B	1.160	0.001	0.403	0.1385
AKAP12	Q02952	A-kinase anchor protein 12	1.144	0.004	2.013	0.0001
TNFAIP8	O95379	Tumor necrosis factor alpha-induced protein 8	1.123	0.042	0.848	0.1045
COMMD8	Q9NX08	COMM domain-containing protein 8	1.116	0.036	-0.304	0.5122
HIST1H3A	P68431	Histone H3.1	1.109	0.014	0.819	0.0497
POLE3	Q9NRF9	DNA polymerase epsilon subunit 3	1.105	0.013	-0.036	0.9200
ASCC1	Q8N9N2	Activating signal cointegrator 1 complex subunit 1	1.083	0.049	0.214	0.6590
BRIX1	Q8TDN6	Ribosome biogenesis protein BRX1 homolog	1.067	0.041	1.706	0.0046
SHCBP1	Q8NEM2	SHC SH2 domain-binding protein 1	1.066	0.034	-0.182	0.7520
SDF2L1	Q9HCN8	Stromal cell-derived factor 2-like protein 1	1.064	0.015	0.317	0.3831
LSM2	Q9Y333	U6 snRNA-associated Sm-like protein LSm2	1.047	0.035	-0.505	0.2566
EIF1B	O60739	Eukaryotic translation initiation factor 1b	1.011	0.012	-0.331	0.3178
HSPH1	Q92598	Heat shock protein 105 kDa	1.011	0.000	0.254	0.0123
TM9SF3	Q9HD45	Transmembrane 9 superfamily member 3	1.010	0.045	0.493	0.2637
BZW2	Q9Y6E2	Basic leucine zipper and W2 domain-containing protein 2	1.008	0.004	0.585	0.0523
FTL	P02792	Ferritin light chain	1.001	0.024	0.318	0.4018
DPH5	Q9H2P9	Diphthine synthase	0.984	0.001	0.356	0.1035
CHORDC1	Q9UHD1	Cysteine and histidine-rich domain-containing protein 1	0.964	0.000	0.418	0.0049
SEC23B	Q15437	Protein transport protein Sec23B	0.949	0.001	0.703	0.0036
HSP90AB2P	Q58FF8	Putative heat shock protein HSP 90-beta 2	0.924	0.000	0.246	0.1543
CLUH	O75153	Clustered mitochondria protein homolog	0.897	0.001	0.653	0.0037
UCK2	Q9BZX2	Uridine-cytidine kinase 2	0.892	0.000	0.040	0.7210
TTC4	O95801	Tetratricopeptide repeat protein 4	0.842	0.001	0.197	0.2589
RACGAP1	Q9H0H5	Rac GTPase-activating protein 1	0.841	0.001	-0.320	0.0794
SUGT1	Q9Y2Z0	Suppressor of G2 allele of SKP1 homolog	0.760	0.000	0.243	0.0393
HSPD1	P10809	60 kDa heat shock protein, mitochondrial	0.710	0.000	-0.036	0.7723
ANLN	Q9NQW6	Actin-binding protein anillin	0.710	0.000	-0.048	0.5340
TMEM263	Q8WUH6	Transmembrane protein 263	0.694	0.001	-0.021	0.8765
TXNRD1	Q16881	Thioredoxin reductase 1, cytoplasmic	0.690	0.000	0.090	0.4401
HSP90AA1	P07900	Heat shock protein HSP 90-alpha	0.687	0.000	-0.141	0.0514
CACYBP	Q9HB71	Calcyclin-binding protein	0.654	0.000	-0.075	0.5152
TNPO1	Q92973	Transportin-1	0.651	0.000	0.304	0.0121
HSPE1	P61604	10 kDa heat shock protein, mitochondrial	0.641	0.000	0.008	0.9439
GSR	P00390	Glutathione reductase, mitochondrial	0.632	0.000	0.291	0.0306
NACA	E9PAV3	Nascent polypeptide-associated complex subunit alpha	0.613	0.000	0.312	0.0140
HSPA8	P11142	Heat shock cognate 71 kDa protein	0.607	0.000	-0.125	0.0633
HSP90AB1	P08238	Heat shock protein HSP 90-beta	0.603	0.000	0.025	0.8044
EIF3I	Q13347	Eukaryotic translation initiation factor 3 subunit I	0.598	0.000	0.032	0.7565
PRDX1	Q06830	Peroxiredoxin-1	0.581	0.000	0.185	0.0690
TBC1D13	Q9NVG8	TBC1 domain family member 13	0.578	0.001	0.249	0.0468
HSPB1	P04792	Heat shock protein beta-1	0.576	0.000	0.344	0.0001
BAG3	O95817	BAG family molecular chaperone regulator 3	0.570	0.000	0.150	0.0448
FKBP4	Q02790	Peptidyl-prolyl cis-trans isomerase FKBP4	0.568	0.000	0.004	0.9664
MAT2A	P31153	S-adenosylmethionine synthase isoform type-2	0.563	0.000	-0.220	0.0184
COTL1	Q14019	Coactosin-like protein	0.531	0.000	0.340	0.0024
STIP1	P31948	Stress-induced-phosphoprotein 1	0.514	0.000	0.009	0.8961
AHNAK	Q09666	Neuroblast differentiation-associated protein AHNAK	-0.505	0.000	-0.180	0.0776
GGH	Q92820	Gamma-glutamyl hydrolase	-0.508	0.001	-0.213	0.0479
CAPG	P40121	Macrophage-capping protein	-0.538	0.000	-0.208	0.0360
PGM1	P36871	Phosphoglucomutase-1	-0.586	0.001	-0.040	0.7137
LSS	P48449	Lanosterol synthase	-0.590	0.001	-0.044	0.7152
AHNAK2	Q8IVF2	Protein AHNAK2	-0.606	0.001	-0.260	0.0596
LRRC47	Q8N1G4	Leucine-rich repeat-containing protein 47	-0.697	0.000	-0.160	0.0673
HNRNPUL2	Q1KMD3	Heterogeneous nuclear ribonucleoprotein U-like protein 2	-0.722	0.001	-0.081	0.5669
IGFBP7	Q16270	Insulin-like growth factor-binding protein 7	-0.968	0.000	-0.128	0.4495
MYLK	Q15746	Myosin light chain kinase, smooth muscle	-0.995	0.001	0.278	0.1802
TGFB2	P61812	Transforming growth factor beta-2	-1.010	0.025	0.329	0.3732
CADM1	Q9BY67	Cell adhesion molecule 1	-1.024	0.031	0.221	0.5888
SORBS2	O94875	Sorbin and SH3 domain-containing protein 2	-1.035	0.000	-0.503	0.0003
DNAH9	Q9NYC9	Dynein heavy chain 9, axonemal	-1.051	0.026	-0.624	0.1429
ARL8B	Q9NVJ2	ADP-ribosylation factor-like protein 8B	-1.053	0.000	-0.081	0.6728
CARKD	Q8IW45	ATP-dependent (S)-NAD(P)H-hydrate dehydratase	-1.127	0.004	-0.596	0.0665
DCLK1	O15075	Serine/threonine-protein kinase DCLK1	-1.162	0.045	-0.634	0.2264
COL5A1	P20908	Collagen alpha-1(V) chain	-1.171	0.004	0.048	0.8710
PCOLCE	Q15113	Procollagen C-endopeptidase enhancer 1	-1.173	0.000	-0.579	0.0143
FYCO1	Q9BQS8	FYVE and coiled-coil domain-containing protein 1	-1.191	0.001	-0.115	0.6204
COL12A1	Q99715	Collagen alpha-1(XII) chain	-1.199	0.002	0.179	0.4323
ANK1	P16157	Ankyrin-1	-1.205	0.001	-0.760	0.0126
MMP14	P50281	Matrix metalloproteinase-14	-1.230	0.005	-0.559	0.0766
SCRIB	Q14160	Protein scribble homolog	-1.269	0.003	-0.600	0.0809
IDS	P22304	Iduronate 2-sulfatase	-1.276	0.030	-1.100	0.0348
TPP1	O14773	Tripeptidyl-peptidase 1	-1.301	0.004	0.141	0.6778
IGFBP3	P17936	Insulin-like growth factor-binding protein 3	-1.336	0.012	-1.506	0.0067
RTF1	Q92541	RNA polymerase-associated protein RTF1 homolog	-1.379	0.027	-0.767	0.1665
ALDH1A3	P47895	Aldehyde dehydrogenase family 1 member A3	-1.390	0.000	-0.977	0.0000
NUCKS1	Q9H1E3	Nuclear ubiquitous casein and cyclin-dependent kinase substrate 1	-1.450	0.006	-0.650	0.1329
ATXN1	P54253	Ataxin-1	-1.613	0.002	-0.524	0.2165
TUBB2B	Q9BVA1	Tubulin beta-2B chain	-1.959	0.004	-1.498	0.0088
HLA-B	P30685	HLA class I histocompatibility antigen	-2.024	0.001	0.594	0.1853
CNPY3	Q9BT09	Protein canopy homolog 3	-2.076	0.013	-1.179	0.0563

B

Gene name	Protein Ref.	Protein name	log2FC NOD2 vs P	pvalue NOD2 vs P	log2FC NOD1 vs P	pvalue NOD1 vs P
NOD2	Q9HC29	Nucleotide-binding oligomerization domain-containing protein 2	2.516	0.0006	-0.504	0.1526
AKAP12	Q02952	A-kinase anchor protein 12	2.013	0.0001	1.144	0.0044
TUBA4A	P68366	Tubulin alpha-4A chain	1.805	0.0000	0.613	0.0029
ZFAND6	Q6FIF0	AN1-type zinc finger protein 6	1.750	0.0163	-0.329	0.2820
RPL6	Q02878	60S ribosomal protein L6	1.709	0.0283	0.695	0.3088
BRIX1	Q8TDN6	Ribosome biogenesis protein BRX1 homolog	1.706	0.0046	1.067	0.0409
ANXA3	P12429	Annexin A3	1.511	0.0002	0.939	0.0035
RPL18	Q07020	60S ribosomal protein L18	1.459	0.0193	0.286	0.5823
RPS27L	Q71UM5	40S ribosomal protein S27-like	1.379	0.0044	-0.332	0.3724
TEX10	Q9NXF1	Testis-expressed sequence 10 protein	1.355	0.0031	0.845	0.0281
MYBBP1A	Q9BQG0	Myb-binding protein 1A	1.345	0.0134	0.624	0.1809
ASS1	P00966	Argininosuccinate synthase	1.304	0.0003	0.034	0.8778
MAPRE2	Q15555	Microtubule-associated protein RP/EB family member 2	1.288	0.0011	0.710	0.0262
RAB1A	P62820	Ras-related protein Rab-1A	1.279	0.0006	0.525	0.0566
H2AFY	O75367	Core histone macro-H2A.1	1.264	0.0003	-0.056	0.7975
RPL36AL	Q969Q0	60S ribosomal protein L36a-like	1.253	0.0421	0.689	0.2202
RSL1D1	O76021	Ribosomal L1 domain-containing protein 1	1.226	0.0100	0.525	0.1887
SURF4	O15260	Surfeit locus protein 4	1.223	0.0472	0.369	0.4921
RPL15	P61313	60S ribosomal protein L15	1.206	0.0497	0.356	0.5152
RPL13A	P40429	60S ribosomal protein L13a	1.198	0.0310	0.287	0.5488
RPL3	P39023	60S ribosomal protein L3	1.162	0.0387	0.494	0.3244
NFKB1	P19838	Nuclear factor NF-kappa-B p105 subunit	1.155	0.0497	0.531	0.3189
PLEKHO2	Q8TD55	Pleckstrin homology domain-containing family O member 2	1.143	0.0004	0.681	0.0087
RPL14	P50914	60S ribosomal protein L14	1.143	0.0143	0.402	0.3042
TPRG1L	Q5T0D9	Tumor protein p63-regulated gene 1-like protein	1.141	0.0117	0.883	0.0212
RPL32	P62910	60S ribosomal protein L32	1.140	0.0473	0.567	0.2776
HIP1	O00291	Huntingtin-interacting protein 1	1.117	0.0143	0.347	0.3543
PMPCB	O75439	Mitochondrial-processing peptidase subunit beta	1.114	0.0010	0.640	0.0195
RPL4	P36578	60S ribosomal protein L4	1.083	0.0178	0.362	0.3500
RPL7	P18124	60S ribosomal protein L7	1.080	0.0045	-0.135	0.6393
NOL7	Q9UMY1	Nucleolar protein 7	1.030	0.0085	0.254	0.4513
SERPINE1	P05121	Plasminogen activator inhibitor 1	1.029	0.0012	-0.124	0.5722
H1FX	Q92522	Histone H1x	1.023	0.0399	0.218	0.6164
LCP1	P13796	Plastin-2	0.772	0.0008	0.388	0.0303
PDLIM4	P50479	PDZ and LIM domain protein 4	0.752	0.0006	0.385	0.0237
MOCS2	O96033	Molybdopterin synthase sulfur carrier subunit	0.729	0.0006	1.178	0.0000
FAHD2A	Q96GK7	Fumarylacetoacetate hydrolase domain-containing protein 2A	0.709	0.0002	0.332	0.0182
NIFK	Q9BYG3	MKI67 FHA domain-interacting nucleolar phosphoprotein	0.709	0.0001	0.481	0.0018
STAT6	P42226	Signal transducer and activator of transcription 6	0.688	0.0008	0.049	0.7189
SORBS2	O94875	Sorbin and SH3 domain-containing protein 2	-0.503	0.0003	-1.035	0.0000
PFKP	Q01813	ATP-dependent 6-phosphofructokinase, platelet type	-0.509	0.0008	-0.179	0.1019
TCEB1	Q15369	Transcription elongation factor B polypeptide 1	-0.515	0.0007	-0.019	0.8490
SNAP29	O95721	Synaptosomal-associated protein 29	-0.549	0.0008	-0.033	0.7655
S100A16	Q96FQ6	Protein S100-A16	-0.615	0.0004	-0.176	0.1329
LGMN	Q99538	Legumain	-0.692	0.0010	-0.451	0.0109
YRDC	Q86U90	YrdC domain-containing protein, mitochondrial	-0.775	0.0009	0.294	0.0877
KCTD12	Q96CX2	BTB/POZ domain-containing protein KCTD12	-0.865	0.0001	-0.619	0.0011
ALDH1A3	P47895	Aldehyde dehydrogenase family 1 member A3	-0.977	0.0000	-1.390	0.0000
CASP3	P42574	Caspase-3	-0.989	0.0000	0.025	0.7944
IDS	P22304	Iduronate 2-sulfatase	-1.100	0.0348	-1.276	0.0304
CNOT4	O95628	CCR4-NOT transcription complex subunit 4	-1.303	0.0179	-0.509	0.1728
KIAA0196	Q12768	WASH complex subunit strumpellin	-1.376	0.0177	-1.073	0.0685
PDLIM3	Q53GG5	PDZ and LIM domain protein 3	-1.460	0.0000	-0.716	0.0028
TUBB2B	Q9BVA1	Tubulin beta-2B chain	-1.498	0.0088	-1.959	0.0039
IGFBP3	P17936	Insulin-like growth factor-binding protein 3	-1.506	0.0067	-1.336	0.0122
SHISA6	Q6ZSJ9	Protein shisa-6 homolog	-1.620	0.0053	-0.205	0.6448
DAG1	Q14118	Dystroglycan	-1.651	0.0039	0.639	0.0100
SVIL	O95425	Supervillin	-1.684	0.0011	-0.409	0.2612
