# Supplementary material for: Proteome profiling of triple negative breast cancer cells overexpressing NOD1 and NOD2 receptors unveils molecular signatures of malignant cell proliferation
Source: BMC Genomics. 2019 Feb 21;20:152. doi: 10.1186/s12864-019-5523-6 (PMC6385390; doi:10.1186/s12864-019-5523-6)
Supplement: Supplementary file 2 — GSEA gene sets Enriched gene sets detected by GSEA in HS578T/NOD1 (A) and HS578T/NOD2 (B) cells. Gene sets are ranked and color coded by their NES (Normalized Enrichment Score) relative to unmodified Hs578T cells (P). For each gene set, name and size of the gene set, ES (Enrichment Score), NES, nominal p-value and FDR (False Discovery Rate) are reported. Color coding performed in MS Office Excel, Red: upregulated. Blue: downregulated. (PDF 215 kb) [file 12864_2019_5523_MOESM2_ESM.pdf]

A

NAME	SIZE	ES	NES	NOM p-val	FDR q-val
KEGG_PYRIMIDINE_METABOLISM	42	0.65745443	2.2660184	0.0000	0.0000
HALLMARK_MYC_TARGETS_V2	40	0.6329294	2.1353428	0.0000	0.0000
HALLMARK_REACTIVE_OXIGEN_SPECIES_PATHWAY	23	0.66056067	1.9783748	0.0000	0.0013
REACTOME_METABOLISM_OF_NUCLEOTIDES	45	0.5610285	1.9362094	0.0017	0.0353
REACTOME_SYNTHESIS_AND_INTERCONVERSION_OF_NUCLEOTIDE_DI_AND_TRIPHOSPHATES	15	0.7161858	1.9228737	0.0017	0.0220
KEGG_NOD_LIKE_RECEPTOR_SIGNALING_PATHWAY	15	0.67985487	1.8278371	0.0018	0.0257
REACTOME_INFLUENZA_LIFE_CYCLE	116	0.42666072	1.7494111	0.0000	0.1390
KEGG_PURINE_METABOLISM	55	0.4781754	1.7409075	0.0015	0.0448
HALLMARK_E2F_TARGETS	109	0.4292668	1.737116	0.0014	0.0177
HALLMARK_UV_RESPONSE_UP	56	0.4653759	1.6977409	0.0048	0.0230
REACTOME_ANTIGEN_PROCESSING_UBIQUITINATION_PROTEASOME_DEGRADATION	89	0.41763365	1.6590726	0.0045	0.2137
KEGG_GLUTATHIONE_METABOLISM	28	0.5250061	1.6410465	0.0133	0.0934
KEGG_UBIQUITIN_MEDIATED_PROTEOLYSIS	42	0.48382598	1.6329755	0.0112	0.0798
HALLMARK_MTORC1_SIGNALING	119	0.38082024	1.5638815	0.0030	0.0763
KEGG_RIBOSOME	75	0.4025802	1.5566938	0.0063	0.1249
HALLMARK_APOPTOSIS	48	0.43056756	1.5198188	0.0223	0.0909
KEGG_SPLICEOSOME	98	0.3708124	1.4939808	0.0030	0.1723
KEGG_DNA_REPLICATION	24	0.4871667	1.4873236	0.0471	0.1588
HALLMARK_G2M_CHECKPOINT	86	0.3694863	1.4590614	0.0286	0.1196
HALLMARK_DNA_REPAIR	69	0.38106045	1.4234469	0.0290	0.1369
HALLMARK_MYC_TARGETS_V1	180	0.3118267	1.3545369	0.0234	0.1936
HALLMARK_ALLOGRAFT_REJECTION	36	-0.40616748	-1.4485587	0.0474	0.0961
KEGG_ECM_RECEPTOR_INTERACTION	24	-0.4563365	-1.4736401	0.0474	0.2388
REACTOME_TRANSMISSION_ACROSS_CHEMICAL_SYNAPSES	26	-0.44953883	-1.4782183	0.0403	0.2076
HALLMARK_INTERFERON_ALPHA_RESPONSE	29	-0.4281655	-1.4785986	0.0420	0.0876
KEGG_CALCIUM_SIGNALING_PATHWAY	19	-0.49786124	-1.5230166	0.0370	0.1875
KEGG_GNRH_SIGNALING_PATHWAY	21	-0.47977847	-1.5288852	0.0337	0.2000
KEGG_RENAL_CELL_CARCINOMA	20	-0.49976796	-1.5343391	0.0389	0.2163
KEGG_AXON_GUIDANCE	24	-0.487177	-1.5383517	0.0336	0.2414
REACTOME_PLATELET_ACTIVATION_SIGNALING_AND_AGGREGATION	57	-0.39981022	-1.5720456	0.0104	0.1297
REACTOME_SEMAPHORIN_INTERACTIONS	24	-0.48854607	-1.5826993	0.0291	0.1320
REACTOME_GPCR_DOWNSTREAM_SIGNALING	28	-0.47128528	-1.5901694	0.0149	0.1387
KEGG_GLYCOLYSIS_GLUCONEOGENESIS	33	-0.4709478	-1.595909	0.0250	0.2292
KEGG_NATURAL_KILLER_CELL_MEDIATED_CYTOTOXICITY	19	-0.5293883	-1.6313763	0.0214	0.2122
REACTOME_PHOSPHOLIPID_METABOLISM	28	-0.4840428	-1.6365699	0.0169	0.1115
REACTOME_DEVELOPMENTAL_BIOLOGY	102	-0.37862894	-1.6476864	0.0000	0.1164
KEGG_REGULATION_OF_ACTIN_CYTOSKELETON	80	-0.3925666	-1.6609098	0.0026	0.2254
HALLMARK_MYOGENESIS	44	-0.45492598	-1.6771286	0.0113	0.0206
REACTOME_G_ALPHA1213_SIGNALLING_EVENTS	16	-0.5798612	-1.6777186	0.0113	0.1061
HALLMARK_HYPOXIA	59	-0.4239711	-1.6815752	0.0027	0.0246
HALLMARK_APICAL_JUNCTION	59	-0.42030552	-1.6821451	0.0110	0.0324
REACTOME_AXON_GUIDANCE	83	-0.41057375	-1.7237672	0.0030	0.0818
REACTOME_NEUROTRANSMITTER_RECEPTOR_BINDING_AND_DOWNSTREAM_TRANSMISSION_IN_THE_POSTSYNAPTIC_CELL	21	-0.57079726	-1.7754589	0.0102	0.0579
HALLMARK_UV_RESPONSE_DN	35	-0.5443186	-1.907133	0.0000	0.0060
REACTOME_NCAM_SIGNALING_FOR_NEURITE_OUT_GROWTH	19	-0.6426916	-1.9113427	0.0000	0.0189
REACTOME_COLLAGEN_FORMATION	18	-0.67500585	-1.9983647	0.0000	0.0100
REACTOME_EXTRACELLULAR_MATRIX_ORGANIZATION	20	-0.66631	-2.05025	0.0000	0.0068
KEGG_LEUKOCYTE_TRANSENDOTHELIAL_MIGRATION	36	-0.5653549	-2.0548573	0.0000	0.0023
KEGG_FOCAL_ADHESION	72	-0.528412	-2.1093404	0.0000	0.0019
HALLMARK_EPITHELIAL_MESENCHYMAL_TRANSITION	77	-0.53468835	-2.2429752	0.0000	0.0000

B

NAME	SIZE	ES	NES	NOM p-val	FDR q-val
REACTOME_PEPTIDE_CHAIN_ELONGATION	76	0.6214367	2.3735838	0	0
KEGG_RIBOSOME	75	0.632319	2.3656912	0	0
REACTOME_NONSENSE_MEDIATED_DECAY_ENHANCED_BY_THE_EXON_JUNCTION_COMPLEX	85	0.5903059	2.2877703	0	0
REACTOME_SRP_DEPENDENT_COTRANSLATIONAL_PROTEIN_TARGETING_TO_MEMBRANE	85	0.5764691	2.2310007	0	0
REACTOME_3_UTR_MEDIATED_TRANSLATIONAL_REGULATION	96	0.5299217	2.0773249	0	4.05E-04
REACTOME_INFLUENZA_VIRAL_RNA_TRANSCRIPTION_AND_REPLICATION	89	0.5065527	1.9796326	0	0.003069731
REACTOME_TRANSLATION	121	0.47664908	1.939665	0	0.004494937
REACTOME_INFLUENZA_LIFE_CYCLE	116	0.45196784	1.8209221	0	0.017631909
HALLMARK_INFLAMMATORY_RESPONSE	21	0.559271	1.629521	0.018644068	0.185041
HALLMARK_MYC_TARGETS_V2	40	0.4758651	1.6158905	0.014308427	0.102411866
REACTOME_METABOLISM_OF_MRNA	172	0.36721912	1.5833695	0.001340483	0.2099004
HALLMARK_CHOLESTEROL_HOMEOSTASIS	27	-0.4887721	-1.610667	0.009756098	0.24410538
